# Screening for abnormal glycosylation in a cohort of adult liver disease patients

**DOI:** 10.1002/jimd.12273

**Published:** 2020-07-17

**Authors:** Jos C. Jansen, Bart van Hoek, Herold J. Metselaar, Aad P. van den Berg, Fokje Zijlstra, Karin Huijben, Monique van Scherpenzeel, Joost P. H. Drenth, Dirk J. Lefeber

**Affiliations:** ^1^ Department of Gastroenterology and Hepatology Radboud University Medical Centre Nijmegen Netherlands; ^2^ Department of Neurology, Translational Metabolic Laboratory Radboud University Medical Centre Nijmegen Netherlands; ^3^ Department of Gastroenterology and Hepatology Leiden University Medical Centre Leiden Netherlands; ^4^ Department of Gastroenterology and Hepatology Erasmus Medical Centre Rotterdam Rotterdam Netherlands; ^5^ Department of Gastroenterology and Hepatology University Medical Centre Groningen Groningen Netherlands

**Keywords:** congenital disorders of glycosylation, end‐stage liver disease, hyperfucosylation, *N*‐glycosylation, V‐ATPase assembly factor defects

## Abstract

Congenital disorders of glycosylation (CDG) are a rapidly expanding group of rare genetic defects in glycosylation. In a novel CDG subgroup of vacuolar‐ATPase (V‐ATPase) assembly defects, various degrees of hepatic injury have been described, including end‐stage liver disease. However, the CDG diagnostic workflow can be complex as liver disease per se may be associated with abnormal glycosylation. Therefore, we collected serum samples of patients with a wide range of liver pathology to study the performance and yield of two CDG screening methods. Our aim was to identify glycosylation patterns that could help to differentiate between primary and secondary glycosylation defects in liver disease. To this end, we analyzed serum samples of 1042 adult liver disease patients. This cohort consisted of 567 liver transplant candidates and 475 chronic liver disease patients. Our workflow consisted of screening for abnormal glycosylation by transferrin isoelectric focusing (tIEF), followed by in‐depth analysis of the abnormal samples with quadruple time‐of‐flight mass spectrometry (QTOF‐MS). Screening with tIEF resulted in identification of 247 (26%) abnormal samples. QTOF‐MS analysis of 110 of those did not reveal glycosylation abnormalities comparable with those seen in V‐ATPase assembly factor defects. However, two patients presented with isolated sialylation deficiency. Fucosylation was significantly increased in liver transplant candidates compared to healthy controls and patients with chronic liver disease. In conclusion, a significant percentage of patients with liver disease presented with abnormal CDG screening results. However, the glycosylation pattern was not indicative for a V‐ATPase assembly factor defect. Advanced glycoanalytical techniques assist in the dissection of secondary and primary glycosylation defects.

## Graphical abstract

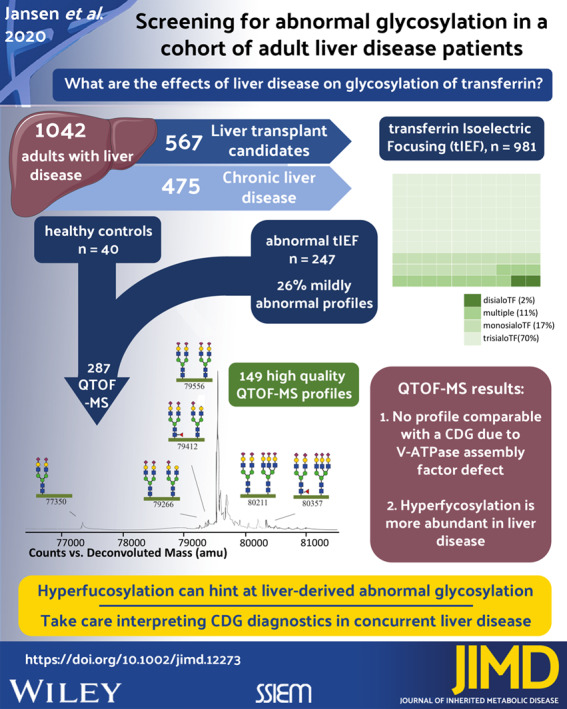

## 1 INTRODUCTION

Congenital disorders of glycosylation (CDG) are a group of inborn errors of metabolism characterized by abnormal glycosylation. Most CDG share a multisystem phenotype with dysmorphic features, failure to thrive, and neurological symptoms.^1^ Involvement of the liver is frequent in CDG but usually not the dominant feature.^2^

In recent years, a novel subgroup of CDG patients has emerged that presents predominantly with a hepatic phenotype.^3^, ^4^, ^5^, ^6^, ^7^ Pathological variants in this group are in genes that code for assembly factors of the vacuolar‐ATPase (V‐ATPase), the proton pump for intracellular acidification. The glycosylation pattern resembles a type 2 CDG with loss of sialic acid and galactose. The hepatic clinical spectrum ranges from mildly elevated serum transaminases and steatosis, resembling nonalcoholic fatty liver disease, to cirrhosis and end‐stage liver disease warranting liver transplantation (LTx).

Liver cirrhosis develops as a response to chronic liver injury. The central pathological event in cirrhosis is deposition of extracellular matrix increasing hepatic flow resistance with ensuing hepatocyte dysfunction.^8^ Patients with cirrhosis have a high risk of decompensation of their liver disease. It can develop in end‐stage liver disease (ESLD), necessitating LTx.

Abnormal glycosylation is a known phenomenon in chronic liver disease and can be used to discriminate between different fibrosis stages and cirrhosis and provides an interesting and noninvasive alternative for liver biopsy.^9^ Abnormal glycan structures of liver‐derived proteins such as transferrin (TF) and haptoglobin have been described in alcoholic liver disease, nonalcoholic steatohepatitis and primary sclerosing cholangitis.^10^, ^11^, ^12^ These abnormalities include hyperfucosylation due to increased fucosyltransferase activity and hyposialylation as a result of lower sialyltransferase activity.

Traditionally, new CDG patients are identified through isoelectric focusing of TF (tIEF).^13^ TF possesses two biantennary glycans at amino acids Asn432 and Asn630, both negatively charged because of the terminal sialic acids.^14^, ^15^ tIEF uses loss of these sialic acids to separate the various isoforms. A disadvantage of tIEF is that it only provides information on desialylation and not for example on hypogalactosylation and fucosylation. The use of quadruple time‐of‐flight mass‐spectrometry (QTOF‐MS) overcomes this disadvantage by providing in‐depth high resolution information on the glycans attached to TF.^16^

The primary aim of this study was to identify glycosylation patterns that could help to differentiate between primary and secondary glycosylation defects in liver disease within a cohort of adult patients with end‐stage liver disease. As a secondary aim, we studied to what extent liver disease affects CDG screening.

## 2 MATERIALS AND METHODS

### 2.1 Selection of liver disease patients, sample selection, and ethical considerations

We collected 1042 samples from patients with an established chronic liver disease from four Dutch tertiary referral hospitals. Samples were collected in the period 1993 to 2013. We specifically targeted ESLD patients who were evaluated and waitlisted for LTx. These samples were provided by Erasmus MC in Rotterdam (n = 264), LUMC in Leiden (n = 142), and UMCG in Groningen (n = 155). All samples were drawn and aliquoted preceding LTx.

Chronic liver disease samples (n = 410), without ESLD, were provided by the Radboudumc in Nijmegen and by the LUMC (n = 65, all with a diagnosis of auto‐immune hepatitis). These patients were seen at the outpatient clinic for diagnosis and treatment of a range of liver diseases. Patients with known viral hepatitis (infectious hepatitis B, C, or E) were excluded from analysis as we hypothesized that the presence of underlying CDG would be unlikely in this patient population. Samples from healthy controls (n = 40) were obtained from the local bloodbank. All samples were stored at −80°C until analysis.

Material was collected in agreement with the Dutch code of conduct for responsible use of human tissue (Dutch Federation of Biomedical Scientific Societies, www.federa.org). All experiments were performed in accordance with the guidelines and regulations of the Ethics Committee of the Radboudumc. Approval was documented in case file 2018‐5012.

### 2.2 Study design and workflow

We first performed tIEF of the collected samples to identify global *N*‐glycosylation defects with hyposialylation. Sixty‐one samples were excluded prior to tIEF: 24 samples because they were drawn after liver transplantation, 21 were of very poor quality and unsuitable for further workup, 5 samples were wrongly allocated, 10 patients were < 18 years of age at time of sampling, and 1 sample was from a patient with an established diagnosis of CDG.

We defined abnormal TF sialylation as an increased percentage of hyposialylated TF isoforms compared with the main isoform, tetrasialoTF. Control ranges were used from the clinical diagnostic protocol, derived by tIEF of 59 healthy control samples: asialoTF 0%‐3.2%; monosialoTF 0%‐5.0%; disialoTF 3.3% to 7.6%; trisialoTF 4.9% to 10.6%; pentasialoTF 18.7% to 31.5%.

If applicable, we designated the profile type 1 or type 2 CDG based on international consensus. Type 1 CDG has increased asialo and disialo TF isoforms, indicating loss of 1 or 2 glycans. Type 2 CDG has hyposialylation for all TF isoforms.^17^

**FIGURE 1:**
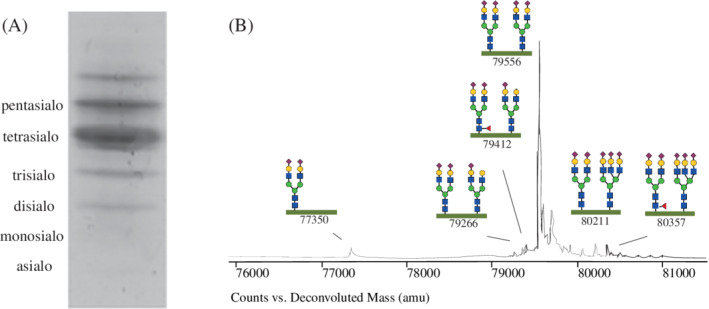
Overview of a normal tIEF and QTOF‐MS profile. A, Typical tIEF pattern. The most abundant fraction correlates with the intact TF glycoprotein. B, Typical QTOF‐MS profile of intact TF with two attached glycans. Shown are the most commonly encountered glycans. The green horizontal bar corresponds with the amino acid backbone. The peak at 79 556 Da correlates with the intact TF glycoprotein. The legend for the monosaccharides is blue square: *N*‐acetylglucosamine, red triangle: fucose, green circle: mannose, yellow circle: galactose, purple diamond: sialic acid

Abnormal tIEF samples were selected for further work‐up with QTOF‐MS. Quality criteria for inclusion of the sample included an abundance of >50 000 of the intact TF glycoprotein (79 556 Da). We selected peaks corresponding to a known TF isoform with an abundance of >1000. The relative abundance of these peaks was calculated based on their percentage relative to total abundance. Figure 1 shows a typical tIEF and QTOF‐MS pattern and depicts the nomenclature of the glycosylation isoforms used throughout this manuscript.

### 2.3 Sample preparation for *N*‐glycan analysis

#### 2.3.1 Transferrin isoelectric focusing

All samples were analyzed with tIEF to determine abnormal sialylation of TF. tIEF was performed as described before.^14^ Briefly, serum or plasma samples were incubated with iron and applied to a 5 to 7 pH gradient gel for electrophoresis. After completion, gels were incubated with 2.5 μL/cm^2^ anti‐TF antibody (Dako #A0061, Carpinteria, CA) and visualized with Coomassie blue. Data analysis was performed with Image Quant Software (Totallab, Newcastle upon Tyne, UK).

TF polymorphisms were recognized by doubled bands for all isoforms. For confirmation, neuraminidase treatment, was applied on samples with ambiguous isoelectric patterns, as described before.^15^

#### 2.3.2 Nano liquid chromatography‐chip (C8)‐quadruple time of flight mass spectrometry (QTOF‐MS)

Mass spectrometry analysis was performed as described before.^16^ First, beads were loaded with anti‐TF antibody (Dako #A0061, Carpinteria, CA) and stored in 20% ethanol. Prior to usage, beads were washed four times with a Tris‐HCl (pH 7) solution. Next, 100 μL of a 1:10 plasma sample dilution in 0.9% sodium chloride was mixed with beads and incubated for 20 minutes under continuous shaking at 3000 rpm/min. Subsequently, beads were washed four times with Tris‐HCl (pH 7) solution. For elution, 1 μL of Tris‐HCl pH 9 solution was added to the sample followed by 50 μL elution buffer (0.1 M Glycine‐HCl pH 2.7). After spinning and verification of neutral pH, 2 μL sample was injected into the microfluidic 6540 HPLC‐chip‐QTOF instrument (Agilent Technologies, Santa Clara, CA). For data analysis of QTOF‐MS profiles, Agilent Mass Hunter Qualitative Analysis software (v. B.04.00) was used.

### 2.4 Statistical analysis

For statistical analysis SPSS Statistics v. 22 (IBM Corporation, Armonk, NY) was used. Due to nonlinearity of our data we used the nonparametric Kruskall‐Wallis test for comparisons of more than two groups and the Mann‐Whitney *U* test when there were two groups. *P*‐values were adjusted for multiple testing with the Bonferroni method.

## 3 RESULTS

**FIGURE 2:**
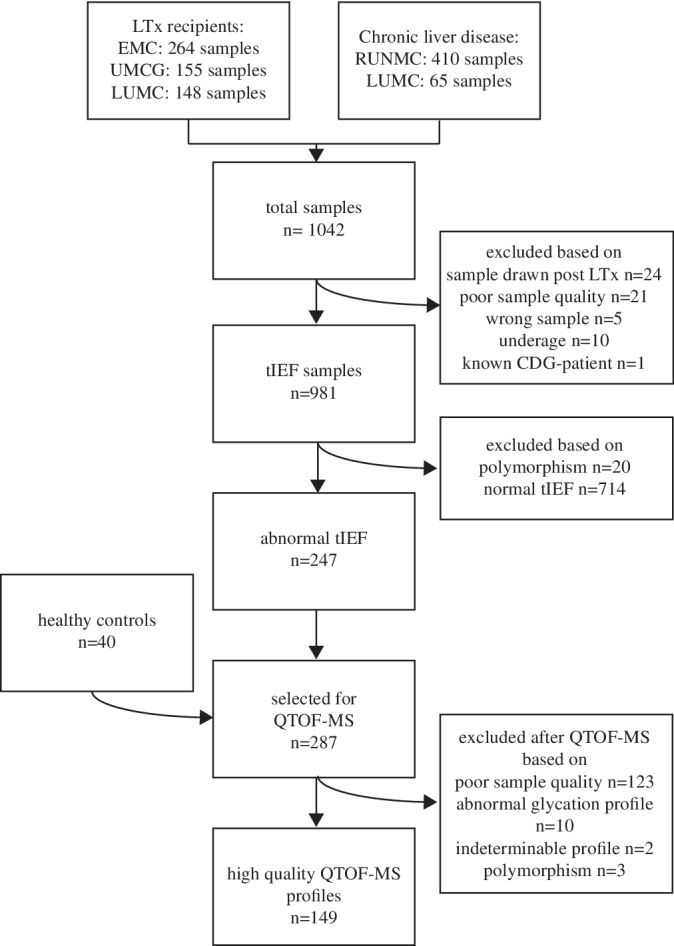
Flowchart of the study design

A total of 1042 samples were collected, 567 samples of LTx candidates and 475 samples of patients with CLD. Figure 2 shows a flowchart of the study design. We included 981 samples for further N‐glycan analysis with tIEF. After tIEF, 744 samples including 20 samples with a confirmed polymorphisms were excluded based on a normal profile.

Subsequently, QTOF‐MS was performed on 247 samples and 40 healthy controls. After QTOF‐MS analysis, 125 samples were excluded because the main peak (at 79 556 Da) was of insufficient abundance, 10 samples were excluded because of an abnormal glycation profile and 3 because of a TF polymorphism (of which 2 were not identified with tIEF and 1 was a healthy control). In total, 110 patient samples and 39 healthy control samples were of sufficient quality for interpretation of the glycosylation profile. Table 1 shows patient characteristics for the selected samples. Table S1 shows tIEF and QTOF‐MS results for all samples with an abnormal tIEF profile.

**TABLE 1:** Patient characteristics

	tIEF selected samples (n = 961)		QTOF‐MS selected samples (n = 149)		
	LTx (n = 511)	CLD (n = 450)	LTx (n = 76)	CLD (n = 34)	HC (n = 39)
Mean age (years)	48.5 (SD 12.2)	50.0 (SD 15.5)	52.6 (SD 12.0)	51.8 (SD 13.2)	47.1 (SD 14.2)
Male sex	283 (55.4%)	211 (46.9%)	50 (65.8%)	15 (44.1%)	21 (53.8%)
Etiology					
Acute liver failure	38 (7.4%)	2 (0.4%)	1 (1.3%)	0	n.a.
Alcoholic liver disease	121 (23.7%)	20 (4.4%)	35 (46.1%)	6 (17.6%)	n.a.
Auto‐immune hepatitis	39 (7.6%)	152 (33.8%)	3 (3.9%)	15 (44.1%)	n.a.
Cholestatic liver disease	157 (30.7%)	42 (9.3%)	11 (14.5%)	0	n.a.
Cryptogenic cirrhosis	54 (10.6%)	20 (4.4%)	10 (13.2%)	1 (2.9%)	n.a.
Metabolic disease	26 (5.1%)	7 (1.6%)	7 (9.2%)	0	n.a.
NASH	10 (2.0%)	36 (8.0%)	0	3 (8.8%)	n.a.
Other	61 (11.9%)	36 (8.0%)	8 (10.5%)	2 (5.9%)	n.a.
Unknown	5 (1.0%)	5 (1.1%)	1 (1.3%)	0	n.a.
Gilbert	—	25 (5.6%)	0	2 (5.9%)	n.a.
Viral hepatitis	—	94 (20.9%)	0	5 (14.7%)	n.a.
DILI	—	11 (2.4%)	0	0	n.a.

Abbreviations: CLD, chronic liver disease; DILI, drug induced liver injury; LTx, liver transplant recipient; HC, healthy controls; n.a., not applicable; NASH, nonalcoholic steatohepatitis.

### 3.1 tIEF screening of 961 samples of liver disease patients shows mild glycosylation abnormalities

**FIGURE 3:**
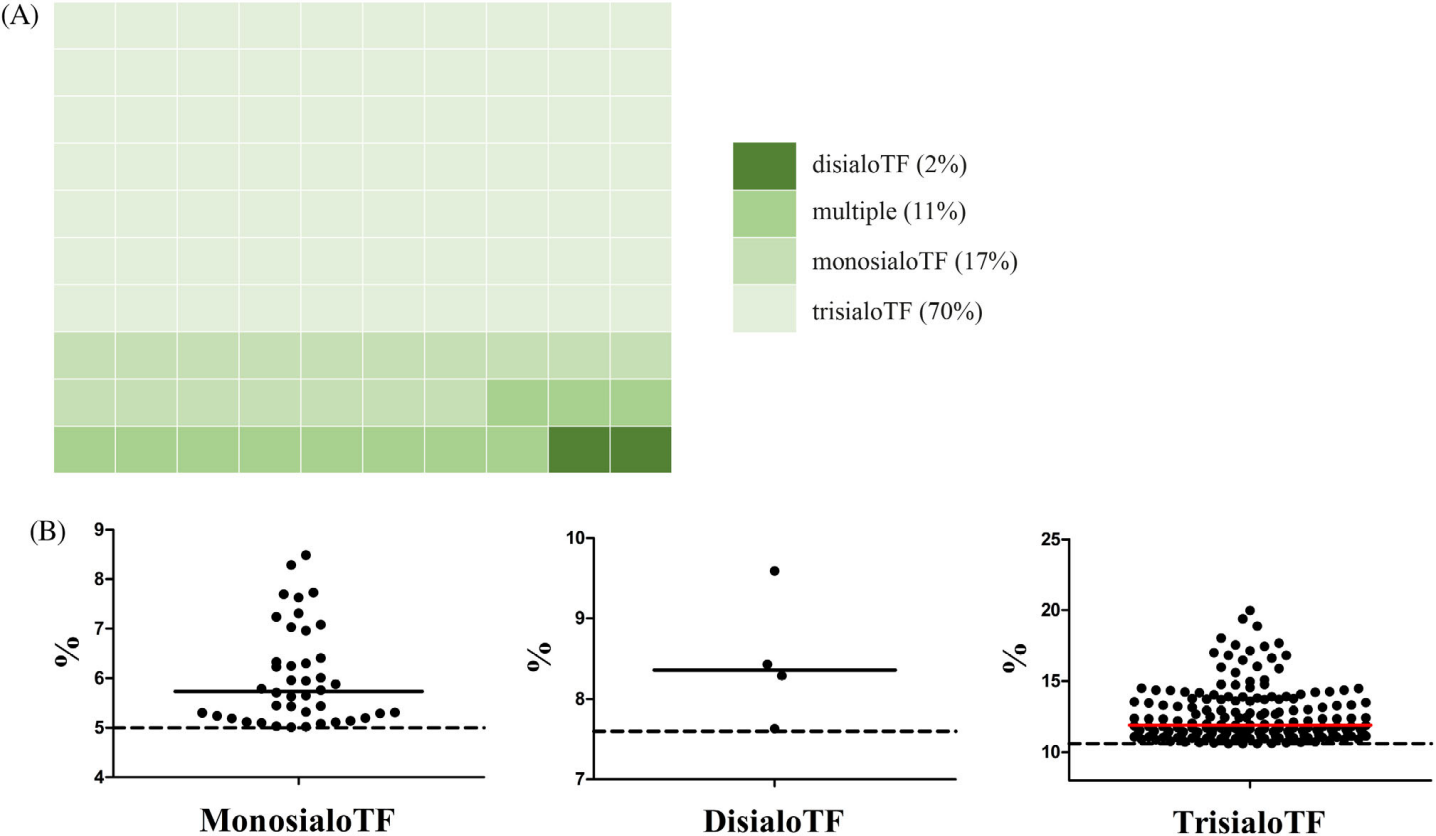
Abnormal tIEF result. A, Waffle chart that shows the distribution of the abnormal tIEF samples. B, Individual medians for the abnormal samples per TF isoform. The dotted line represents upper limit of normal based on internal standards

Out of 961 samples we analyzed using tIEF, 247 samples (26%) had hyposialylation compared to our controls. Of these samples, 175 (70%) had a solitary increased percentage of the trisialoTF isoform, 42 (17%) had an elevated percentage of monosialoTF isoform and 4 (2%) had an elevated percentage of the disialoTF isoform. Mixed combinations of elevated isoforms occurred in 26 (11%) samples (Figure 3A). None of the samples had increased percentages of all isoforms. One sample had slightly increased percentages of the asialo and the disialoTF isoform but did not reach the values to be suggestive for type 1 CDG. The mixed profiles can be compatible with a CDG type 2 profile. However, the increase in percentage is subtle for most samples (Figure 3B).

### 3.2 QTOF‐MS analysis of preselected liver disease patients did not identify profiles compatible with V‐ATPase assembly factor defects

Of the 110 high quality QTOF‐MS profiles, none had a distinguishable type 1 CDG pattern. The peak associated with nonglycosylated TF (peak 75 140) was only present in one LTx patient with progressive familial intrahepatic cholestasis syndrome (1.2% of total glycan abundance), one CLD patient with auto‐immune hepatitis (1.1%), and three HC (all <1% total glycan abundance). Both patients had a normal percentage of asialoTF in tIEF screening and the six patients with an elevated asialoTF fraction with tIEF analysis did not have a detectable peak with mass 75 140, corresponding to nonglycosylated transferrin. Peak 77 350, which corresponds to loss of one glycan, was present in all but five samples and was not significantly different among the groups (data not shown). We did not identify samples that showed typical glycan structural abnormalities that are seen in type 2 CDG profiles and did not identify a pattern compatible with a V‐ATPase assembly factor defect.

We identified two samples (2/110 = 1.8%) with clearly elevated trisialo TF isoform abundance. The first patient in the CLD group was a female, age 55 at sampling, who was seen at the outpatient clinic for hepatic steatosis. Her profile showed an elevated trisialo TF isoform of 15.0% (median for the CLD group is 2.0%) and presence of an additional isoform (mass 78 976 Da, corresponding with loss of 2 sialic acids). There were no signs of other glycosylation abnormalities. The second patient was a male LTx candidate diagnosed with alpha‐1 anti‐trypsin deficiency. At age of sampling, he was 28 years old. His QTOF‐MS profile showed an increase in the trisialo TF isoform of 12.4% (median for the LTx group is 1.9%) and also an increased loss of 2 sialic acids (mass 78 976 Da). For both patients, we could not detect transferrin glycoforms with missing galactose residues, as for example seen in the V‐ATPase assembly defects.

### 3.3 tIEF but not QTOF‐MS shows that loss of one sialic acid is more frequent and more severe in LTx candidates compared to CLD patients and HC

To see what the effect is of liver disease on desialylation, we first compared the tIEF screening results between LTx and CLD samples. Table 2 shows the medians for all isoforms. The prevalence of several isoforms was statistically significantly different between these groups, but medians were within the normal range. Only the LTx group had a median of the trisialo TF isoform above the upper limit of normal and significantly higher than in the chronic liver disease group (11.8% vs 10.4%, *P* < 0.001). An abnormal tIEF pattern was more frequently seen in LTx candidates compared to CLD patients (175/511 = 34% vs 72/450 = 16%, Chi square *P* value = .000).

**TABLE 2:** medians of the different tiEF TF isoforms

		Abnormal samples (n = 247)				
		CLD (n = 72)	LTx (n = 175)		MWU	
TF isoform	Range (%)	Median (%)	SD	Median (%)	SD	*P* value
Asialo	0.0‐3.2	0.94	1.02	1.24	0.79	.001
Mono	0.0‐5.0	4.84	2.52	2.02	1.74	.000
Di	3.3‐7.6	5.25	1.21	5.27	1.39	.588
Tri	4.9‐10.6	10.40	3.07	11.84	2.25	<.001
Tetra	47.3‐62.7	52.63	6.71	52.67	5.72	.476
Penta	18.7‐31.5	20.78	3.94	19.95	3.44	.038

Abbreviations: CLD, chronic liver disease, LTx, liver transplantation, MWU: Mann‐Whitney *U* test; TF, transferrin.

Next, we aimed to gain more insight in desialylation with QTOF‐MS. QTOF‐MS provides additional detail on glycan composition. To investigate desialylation, we used the combined abundance of the trisialo and the fucosylated trisialo TF isoform. Comparison of these combined peaks among the three groups only showed a slight statistically significant difference between the LTx and CLD groups (Table S2). However, because of the broad SDs, we conclude that overall desialylation is not different between LTx candidates, CLD patients, and healthy controls. In conclusion, based on these data, desialylation is more prominent in LTx candidates when measured with tIEF, but these data are not reproduced with QTOF‐MS.

### 3.4 Hyperfucosylation is more pronounced in end‐stage liver disease than in chronic liver disease

**FIGURE 4:**
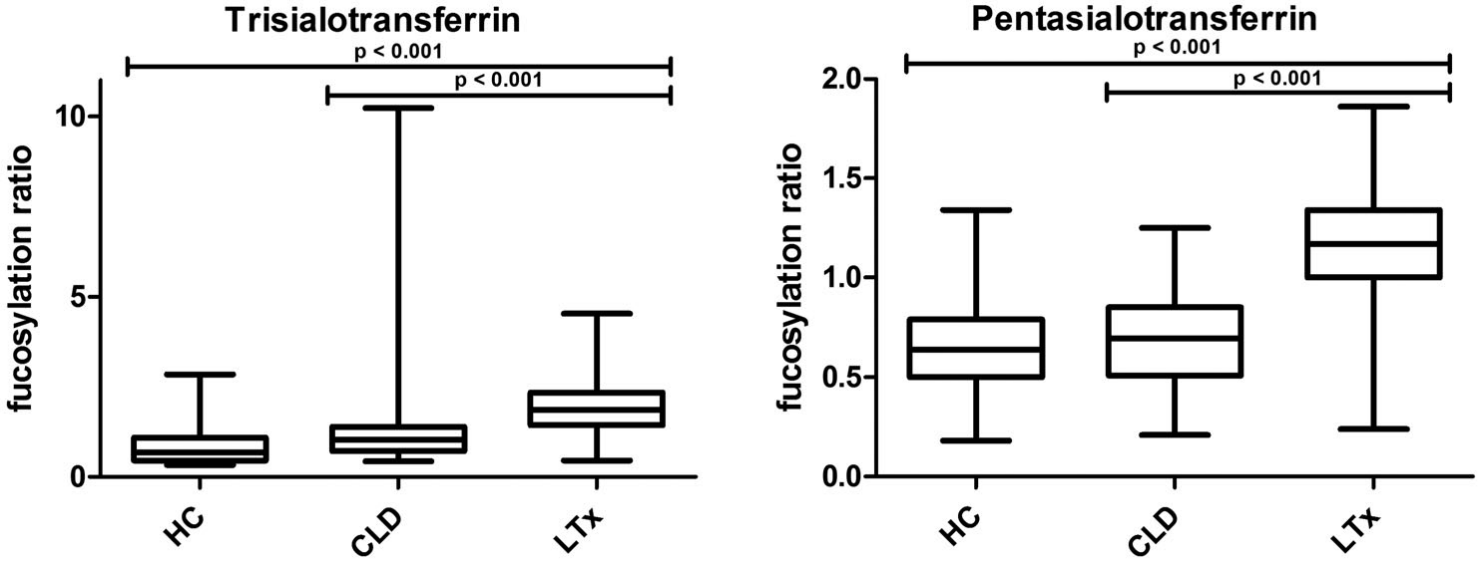
Boxplots of the fucosylation ratio of the tri‐ and pentasialoTF QTOF‐MS isoforms. The left graph shows the fucosylation ratio of trisialotransferrin, or peak 79 266 Da. The right graph shows the fucosylation ratio of pentasialotransferrin, or peak 80 211 Da. We used a Kruskal‐Wallis test for calculation of *P* values. CLD, chronic liver disease; HC, healthy controls; LTx, liver transplantation

Hyperfucosylation of liver derived proteins is a known phenomenon in a variety of liver diseases. Therefore, we calculated the fucosylation ratio for the trisialo and pentasialo TF isoforms (Figure 4 and Table S2). Fucosylation of the tetrasialo TF isoform was not reliably detectable because of overlap with other nearby peaks. Figure 4 shows that the ratios are higher for the LTx samples compared to the HC and the CLD samples (*P* < .0001 for both). These data indicate that hyperfucosylation of TF is more pronounced in end‐stage liver disease compared to chronic liver disease and healthy controls.

## 4 DISCUSSION

In our retrospective analysis of a cohort of 1042 liver disease patients, we found a significant percentage of patients with altered CDG screening results by serum transferrin isoelectric‐focusing. However, we did not find clear evidence for the presence of a CDG due to V‐ATPase assembly factor defects. We found that hyperfucosylation is more pronounced in LTx patients compared to those with milder liver disease and healthy controls, thereby showing that high‐resolution mass spectrometry aids the discrimination between primary and secondary glycosylation abnormalities in CDG screening.

### 4.1 Screening for possible primary glycosylation defects

Initial screening with tIEF resulted in identification of 247 (26%), mostly mild, abnormal patterns. We did not identify a typical CDG type 1 pattern, which we anticipated, as to date there is no known type 1 CDG associated with a mild hepatic phenotype.^1^ The majority of samples had an increase in the trisialo TF isoform. None had an increase of all isoforms, a feature of most type 2 CDG. However, based on these tIEF results, we could not rule out a V‐ATPase assembly factor deficiency as mild abnormalities have been described. QTOF‐MS analysis did not confirm desialylation as seen in tIEF and did not show patterns compatible with type 1 CDG or V‐ATPase assembly factor deficiencies. Similarly, no samples with loss of galactose were observed. Therefore, we conclude that our analysis did not identify novel patients with a V‐ATPase assembly factor defect.

A possible reason for this might be that prevalence is too low for detection within our cohort. The exact prevalence of the whole group of CDG is unknown, but is estimated at 0.1 to 0.5/100.000 in Europe. This number is based on recent data from the European CDG network that estimated that there is a total number of 2500 recognized CDG patients in Europe.^1^ However, these observations are based on patients with a multisystem phenotype. The discovery of the V‐ATPase assembly defect subgroup with a very mild hepatic phenotype could indicate that the prevalence is much higher. We speculated that this subgroup would be enriched in a population that covers the full spectrum of liver disease severity, but no cases were detected.

Another reason might be that glycosylation defects of CDG patients who survive into adulthood can become milder, or even normalize, over time.^18^, ^19^, ^20^ Spontaneous normalization of the glycosylation profile has not yet been investigated for CDG due to V‐ATPase assembly defect. However, spontaneous normalization of serum transaminases has been described.^4^, ^5^, ^6^

We identified two patients with an abnormally high percentage of the isoform with loss of one sialic acid. The reason for this abnormal pattern remains unknown. Glycosylation defects in SLC35A1‐CDG (OMIM 603585), a defect in the CMP‐sialic acid transporter, show pure desialylation effects.^16^, ^21^ However, the phenotype of SLC35A1‐CDG is mostly neurological with dysmorphic features, not hepatic.

Also, the presence of bacterial sialidase in serum can lead to hyposialylation of TF. This has been described for *Streptococcus pneumoniae*‐associated hemolytic ureum syndrome.^22^ However, hemolytic uremic syndrome is primarily a disease of infancy and early childhood and the incidence in the adult population is extremely low.

Another option is that these patients have an as yet unrecognized type of CDG. Our study design did not allow us to further investigate this as we did not have access to fresh plasma, fibroblasts or parental DNA to perform adequate genetic analyses and functional studies.

### 4.2 Glycosylation defects in liver disease

Desialylation has been mostly studied in the context of alcoholic liver disease. Indeed, the carbohydrate deficient transferrin (CDT) test to identify chronic alcohol intake is based on hyposialylation of transferrin. Analysis of CDT in abstaining patients with various degrees of liver disease shows a correlation of high CDT percentages with the Child‐Pugh score.^23^

The pathophysiological mechanism behind alcohol‐induced hypoglycosylation is not fully elucidated. Some studies suggest a primary ER defect.^24^, ^25^ Other studies suggest an effect on the Golgi apparatus.^26^, ^27^ One study investigated gene expression of glycosylation genes in NASH, but found mixed results with upregulation of *ST6GAL2* and downregulation of *ST6GAL1*.^11^

Hyperfucosylation of liver‐derived proteins has been most extensively studied in hepatocellular carcinoma (HCC) patients.^28^, ^29^, ^30^, ^31^ Indeed, fucosylated alpha‐fetoprotein is an established disease marker for HCC in the setting of cirrhosis.^32^ Here, we show that fucosylation of transferrin is elevated in samples from LTx candidates compared to CLD patients and healthy controls.

Hyperfucosylation of transferrin was shown to be the cause of chromatographic abnormalities in CDT testing, or so‐called di‐tri‐bridging (i.e., poor resolution of disialoTF from trisialoTF).^33^ Di‐tri‐bridging is associated with liver disease and is more frequent in cirrhotics than in noncirrhotics.^34^

Somewhat older data exists on fucosylation of haptoglobin in liver disease and shows hyperfucosylation in patients with alcoholic liver disease and primary biliary cholangitis.^35^ A more recent paper showed increased hyperfucosylated glycans on haptoglobin in HCC as well as in cirrhosis.^36^

Previous work on serum glycan analysis in liver cirrhosis identified increased hypogalactosylation and increased modification of the serum *N*‐glycome with a bisecting *N*‐acetylglucosamine.^9^ Log ratios of different glycans could discriminate between early fibrosis and cirrhosis. A follow‐up study showed that undergalactosylation was due to the IgG‐derived glycan fraction.^37^ This is in line with our data that did not show undergalactosylation in any sample. Also, no glycans were observed within our cohort with a bisecting *N*‐acetylglucosamine. This might be explained by protein specific glycosylation.

### 4.3 Strengths and limitations

One strength of our study was that we included a wide range of liver disease patients for analysis. We took care to include patients with mild liver disease but also those with advanced chronic liver disease in need for LTx. Our efforts led to the establishment of a large sample size of over 1000 liver disease patients which adds to the robustness of the study. Additionally, we were able to screen these samples with high resolution mass spectrometry that provided in depth analysis of the intact transferrin glycoprotein, including fucosylation, loss of galactose, and the absence of complete glycans.

A limitation of our study is because of its retrospective character. We had access to collected serum samples but were not in possession of fresh plasma or fibroblasts of patients to perform next generation sequencing, run a CDG‐panel, or perform functional studies. We detected two patients with an increased trisialoTF isoform. Although the cause for this elevation is unknown, we believe that the QTOF‐MS analysis ruled out abnormal glycosylation due to a V‐ATPase assembly factor defect. However, we cannot completely exclude another, possibly novel, CDG.

Another limitation of our study is the high percentage of exclusion of samples resulting from low abundance of serum TF in the samples. A possible explanation could be that low serum TF is associated with cirrhosis.^38^, ^39^ Additionally, the effect of long‐term storage on stability of TF is unknown.

### 4.4 Recommendations

Differentiating primary from secondary glycosylation defects in patients with liver dysfunction can be challenging. Previously analysis of total plasma *N*‐glycans suggested that hyperfucosylation was increased in a single liver disease patient but not in primary CDG.^14^ This study expands these findings to a large patient group. A transferrin hyperfucosylation pattern could guide clinicians in decision making.

Our current research exposes caveats in tIEF as a primary diagnostic step in CDG screening. We show that 26% of tiEF samples were abnormal, but these samples did not show a clear type 2 CDG pattern upon QTOF‐MS analysis. An important message is that tIEF screening can be false positive because of liver dysfunction.

We suggest caution when interpreting tIEF result in patients with a suspected CDG and liver disease and recommend a low‐threshold to use advanced glycoanalytic methods, preferably in an expertise center. A clear hyperfucosylated pattern is more suggestive for a secondary cause and loss of galactose more suggestive for a primary glycosylation defect. When only sialylation is decreased we suggest a repeat sample to rule out the involvement of exogeneous sialidase and a critical review of the phenotype to rule out a CDG with known hyposialylation.

## 5 CONCLUSION

Our screening study did not identify V‐ATPase assembly factor defects in a cohort of severe liver disease, but we show that end‐stage liver disease is associated with hyperfucosylation of transferrin. We confirm that regular CDG screening with tIEF can be complicated by liver disease, itself associated with mildly abnormal tIEF profiles.

## CONFLICT OF INTEREST

The authors declare no potential conflict of interest.

## INFORMED CONSENT

Approval was granted by the Ethics Committee of the Radboudumc and documented in case file 2018‐5012. All procedures followed were in accordance with the ethical standards of the responsible committee on human experimentation (institutional and national) and with the Helsinki Declaration of 1975, as revised in 2000.

## ANIMAL RIGHTS

This article does not contain any studies with human or animal subjects performed by the any of the authors.

## Supporting information


**Table S1** Supporting information

**Table S2** Medians of different TF isoforms measured with QTOF‐MS
