# Supplementary material for: Screening for abnormal glycosylation in a cohort of adult liver disease patients
Source: J Inherit Metab Dis. 2020 Jul 17;43(6):1310–20. doi: 10.1002/jimd.12273 (PMC7689844; doi:10.1002/jimd.12273)
Supplement: Supplementary file 2 — Table S2 Medians of different TF isoforms measured with QTOF‐MS [file JIMD-43-1310-s002.docx]

| **Supplementary Table 2. Medians of different TF isoforms measured with QTOF-MS** | | | | | | | | |
| --- | --- | --- | --- | --- | --- | --- | --- | --- |
|  |  | HC (n=39) | CLD (n=34) | LTx (n=76) | K-W test | HC/CLD | HC/LTx | CLD/LTx |
| Peaks (amu) | structure | Median (SD) | Median (SD) | Median (SD) |  |  |  |  |
| 79556 |  | 72.9 (6.3) | 78.8 (4.9) | 67.9 (7.3) | <0.001 | 0.006 | 0.040 | <0.001 |
| 79266 |  | 3.0 (1.1) | 2.0 (2.5) | 1.9 (1.6) | <0.001 | 0.034 | <0.001 | 0.918 |
| 79412 |  | 1.9 (0.8) | 1.9 (1.7) | 3.3 (1.6) | <0.001 | 1.0 | <0.001 | <0.001 |
| 79266+  79412 |  | 5.2 (1.2) | 4.0 (2.7) | 5.2 (2.0) | 0.029 | 0.273 | 1.0 | 0.024 |
| 80211 |  | 6.1 (1.3) | 8.7 (1.9) | 5.1 (1.4) | <0.001 | <0.001 | 0.007 | <0.001 |
| 80357 |  | 3.5 (1.1) | 5.8 (1.75) | 5.8 (1.4) | <0.001 | <0.001 | <0.001 | 1.0 |
| **Fucosylation ratio** | | | | | | | | |
| Trisialo |  | 0.7 (0.6) | 1.0 (1.9) | 1.8 (1.0) | <0.001 | 1.0 | <0.001 | <0.001 |
| Penta  sialo |  | 0.6 (0.3) | 0.7 (0.2) | 1.2 (0.3) | <0.001 | 1.0 | <0.001 | <0.001 |
| HC=healthy controls, CLD=chronic liver disease, LTx=liver transplantation, K-W=Kruskal Wallis test, SD=standard deviation | | | | | | | | |
